# Determination of the Most Efficient Household Technique for the Reduction of Pesticide Residues from Raw Fish Muscles

**DOI:** 10.3390/foods11091254

**Published:** 2022-04-27

**Authors:** Md. Ariful Islam, S. M. Nurul Amin, Christopher L. Brown, Abdul Shukor Juraimi, Md. Kamal Uddin, Aziz Arshad

**Affiliations:** 1 Department of Aquaculture, Faculty of Agriculture, Universiti Putra Malaysia (UPM), Seri Kembangan 43400, Malaysia; arifulbau@gmail.com (M.A.I.); smnabd@gmail.com (S.M.N.A.); 2 Bangladesh Fisheries Research Institute (BFRI), Shrimp Research Station, Bagerhat 9300, Bangladesh; 3 Department of Aquaculture, FAO World Fisheries University, Pukyong National University, Busan 48547, Korea; brownchristopher38@gmail.com; 4 Department of Crop Science, Faculty of Agriculture, Universiti Putra Malaysia (UPM), Seri Kembangan 43400, Malaysia; ashukur@upm.edu.my; 5 Department of Land Management, Faculty of Agriculture, Universiti Putra Malaysia (UPM), Seri Kembangan 43400, Malaysia; mkuddin@upm.edu.my

**Keywords:** pesticides, raw fish muscle, household treatments, GC-ECD, decontamination, food safety

## Abstract

Substantial quantities of pesticides are routinely applied to enhance agricultural crop production. Pesticides used in this way continuously accumulate in the environment and in foods. Harvested crops contain pesticide residues at various concentrations, with potential harmful impacts on human health. Hence, it is of value to identify techniques for the effective decontamination of tainted foods. However, cleaning with water or household agents, e.g., acetic acid and sodium bicarbonate, are recognized treatments for the efficient degradation of pesticides from vegetables and fruits. There is an apparent void of information about the decontamination treatments for raw fishes using household agents that are affordable for all classes of consumers. Hence, the present study was performed to determine the most efficient household technique for reducing pesticide residue levels from precooked raw fish to ensure the utmost food safety. Fish muscles of four species of fishes, viz., *Clarias gariepinus*, *Channa striatus*, *Anabas testudineus* and *Trichogaster trichopterus*, were treated with six treatments: washing with running tap water (T_1_), dipping in normal water (T_2_), dipping in 2% salt solution (T_3_), dipping in 2% vinegar (T_4_), dipping in 0.1% sodium bicarbonate solution (T_5_) as well as dipping in 0.1% sodium bicarbonate solution + 2% vinegar + 2% salt solution + lemon juice (T_6_), as fish muscle is the major consumable portion of fish. The current study demonstrated that the removal percentage of lindane, heptachlor, aldrin, endosulfan, dieldrin, endrin, DDT, methoxychlor and cypermethrin residues against the treated household treatments, in downward order, were soaking in 0.1% sodium bicarbonate solution + 2% vinegar + 2% salt solution + lemon juice solution (T_6_) > soaking in 2% vinegar (T_4_) solution > soaking in 0.1% sodium bicarbonate (T_5_) solution > soaking in 2% salt (T_3_) solution > washing with running tap water (T_1_) > soaking in stable normal water (T_2_). The treatment of raw fish muscle samples by soaking them in 0.1% sodium bicarbonate solution + 2% vinegar + 2% salt solution + lemon juice was found to be the most efficient household treatment, performing significant reductions (%) in pesticide concentration: 72–80% (*p* < 0.05) in *Channa striata*, 71–79% (*p* < 0.05) in *Clarias gariepinus*, 74–80% (*p* < 0.05) in *Anabas testudineus* as well as 78–81% (*p* < 0.05) in *Trichogaster trichopterus* before cooking.

## 1. Introduction

Pesticides, including insecticides and fungicides, are applied universally for the protection of food origin resources from their surrounding enemies [1]. However, their overused or misused application contributes to extensive environmental contamination, particularly in developing countries. Many outdated, illegal, highly toxic and low-priced chemicals are used ubiquitously in developing states, causing severe acute health problems worldwide [2]. Moreover, only small amounts of all the used pesticides are directly involved in pest control, and excessive amounts of pesticides, routinely applied, can become ‘residues’ in the environment. These contaminants can enter terrestrial and aquatic food chains, where they sustain their concentration and create active, durable and detrimental health effects [1]. From waterbodies surrounding the rice field areas of Peninsular Malaysia, the presence of DDT has been witnessed in Snakehead fish (*Channa striatus*), Javanese carp (*Puntius gonionotus*), gourami (*Trichogaster* sp.) and climbing perch (*Anabas testudineus*) at levels of 0.8–3.6, 1.7–3.6, 2.2 and 0.7–1.2 ng/g, respectively. Endosulfan concentration has also been reported in snakehead (*Channa striatus*), Javanese carp (*Puntius gonionotus*), gourami (*Trichogaster* sp.) and climbing perch (*Anabas testudineus*) at levels of 0.8–4.8, 1.1–4.8, 0.4–3.9 and 0.4–4.2 ng/g, respectively, from the Peninsular Malaysian rice field waterbodies [3]. The concentration of aldrin, DDT, dieldrin, endosulfan, endrin and lindane were also evidenced in marine catfish (*Arius* sp.) and jew fish (*Pennahia* sp.) from the Straits of Malacca region of Malaysia [4]. The concentration of heptachlor was also obtained in catfish (*Arius* sp.), blood cockle (*Anadara granosa*) and mullet (*Valamugil* sp.) from the same area [4]. These reports indicate the evidence of organochlorine pesticide (OCP) residues’ presence in the waterbodies of rice fields’ surrounding areas in Malaysia. Food adulterated with toxic pesticides is associated with serious effects on human health. The universal presence of organochlorine pesticide residues (OCPs) in food has arisen due to their wide-ranging agricultural practices and to being discharged to the environment. Hence, it is justifiable to scrutinize the procedures that focus on the status of food safety, particularly for developing nations where pesticide contamination is ubiquitous due to their unsystematic usage. Hence, it is important to determine uncomplicated, cost-effective approaches for indigent people to strengthen food safety from the noxious pesticides. Food treatment at the household and industrial levels has the potential to present an acceptable means of overcoming issues of unsafe pesticide-contaminated foods [1].

Food safety is a concern that is arising universally because of its direct relationship with human health, where the broad application of noxious pesticides used for controlling pests poses threats. It is, therefore, of value for consumers to learn the particular possibilities of and initiatives regarding how to minimize pesticide intake from their foods. Food treatment is a way of developing food items to a more edible and safe form before their cooking or consumption. The household treatment of food items may lead to reductions in pesticide residues found in raw agricultural commodities [5].

Considerable studies reveal that, in most decontamination treatment cases of food items, washing and household-agent operations would lead to the significant removal of residue levels from food [6,7,8]. The tradition of using primary household agents to eliminate pesticide residues before consumption or cooking has long been practiced. The washing of vegetables or fruits with tap water is a treatment process which has been in practice for a long time because it can be easily performed with usable plain water as well as with formulated solutions of accessible household agents [9]. Salt, baking soda, distilled vinegar, etc., have been among the suggested ingredients for the purpose of decontaminating foods from pesticide residues [10].

Before any treatment, washing foodstuffs in water may lead to increases in the efficiency of the pesticide residues’ decontamination treatments [11,12]. Treatment with 0.1% acetic acid for 15 min significantly reduces pesticides in Chinese kale [13]. The treatment such as dipping food in an acetic acid solution has potential for decontaminating the residues of organochlorine pesticides such as hexachlorobenzene (HCB), lindane and DDT in potatoes. The treatment of cabbage by dipping it in 10% acetic acid for 20 min can remove pesticides such as chlorpyrifos, chlorothalonil, p, p-DDT and cypermethrin to a significant degree [14]. Washing hot peppers, sweet peppers as well as eggplants with 2% acetic acid solution for 1 min reduced profenofos residues [15]. Hence, it is evidenced that acetic acid solutions are a potential, accessible and ready-to-use chemical ingredient which actively decontaminates foodstuffs from the existing pesticide residues before cooking [16].

Various concentrations of salt or NaCl solutions (neutral pH) have been documented to potentially eliminate pesticide residues from raw foods [16]. The treatment of food with 0.9% NaCl for 15 min eliminated carbaryl and methomyl pesticide residues from Chinese kale [9]. Treating hot pepper, sweet pepper and eggplant surfaces with 1% NaCl solution for 1 min delaminated profenofos pesticide residue [15]. An analysis of potatoes with 10% NaCl led to the decontamination of pesticides such as HCB, lindane, DDT, dimethoate, pirimiphos-methyl and malathion [7], as well as chlorpyrifos, chlorothalonil, cypermethrin and p, p-DDT residues in cabbage [14]. Soaking of brinjal in 0.1% sodium bicarbonate (NaHCO_3_) for 10 min may decontaminate pesticides residues of chlorpyrifos, quinalphos, profenophos, cyhalothrin and malathion [17]. Soaking okra with 5% sodium bicarbonate solution for 30 min reduced cypermethrin residue noticeably [18]. The residue of pesticides such as cypermethrin and profenofos are decontaminated from Chinese kale when it is soaked with half of a teaspoon of vinegar in 5 L of water for 10 min [19]. The treatment of fruits with lemon juice also evidently reduces the residues of monocrotophos, fenitrothion and fenvalerate by a noticeable percentage [20].

However, amid the abundance of vegetable decontamination procedures, there are no previous studies on pesticide residue reduction from precooked raw fish muscles using household agents that are easily affordable for all classes of consumers. Several modern technologies, such as irradiation, ultrasonication and ozonation, are being practiced for the decontamination of fish fillets by industrialists and other commercial food processors [21], but those are not easily affordable for general consumers. Although, after cooking, a considerable amount of pesticide residues are reduced in contaminated fishes, the reduction level is not 100%, which may threaten the consumers’ food safety.

Hence, the aim of this study was to determine the most efficient household technique to reduce pesticide residues in precooked raw fish muscle, so as to ensure its food safety.

## 2. Materials and Methods

### 2.1. Chemicals and Reagents

Analytical standards of heptachlor, aldrin, endosulfan, dieldrin, endrin, 4,4-DDT, methoxychlor, lindane and cypermethrin were obtained from Sigma-Aldrich, PESTINAL brand, (Saint Louis, MO, USA), through the supplier JM Instrument and Chemical Supply, Sdn. Bhd (Kajang, Selangor, Malaysia). The purity of each of those pesticide standards was more than 98.0%. High-performance liquid chromatography (HPLC)-grade n-hexane (98% purity) from Thermo Fisher (Bremen, Germany), analytical-grade acetonitrile (MeCN) with 99.8% purity, methanol (98% purity), sodium chloride (NaCl) of 98% purity and anhydrous magnesium sulphate (MgSO_4_) with 98% purity and primary secondary amine (PSA) with a purity of 99% were obtained from Merck (Darmstadt, Germany) through the supplier AS Scientific Supplies Sdn. Bhd (Petaling Jaya, Selangor, Malaysia).

### 2.2. Sampling of Fishes

A total of 70 fish samples were collected from Sekinchan, Sungai Besar, Malaysia. The species collection included: 7 African catfish (*Clarias* *gariepinus*), 7 snakeheads (*Channa striatus*)*,* 21 climbing perch (*Anabas testudineus*) and 35 gouramis (*Trichogaster trichopterus*). All fishes were collected from the paddy-cultivating-area waterbodies of Sungai Besar, Sekinchan, Malaysia. The samples were packed in polyethylene plastic zipped bags, and sealed and labeled properly. The samples were transported to the laboratory and stored at −20 °C until the extraction was undertaken. Muscle samples were collected from the ventral section of each fish. The muscle tissues of African catfish were without skin and the muscle tissues of other species were without scales.

### 2.3. Treatment of the Raw Fishes with Pesticides and Household Decontamination Methods: Analysis

An amount of 10 g of raw fish muscle sample from each species was weighed on the analytical balance for each household treatment and the untreated control containing three replications. Then, each of the 10 g fish muscle samples was spiked with 0.5 ppm (500 µg/g) of mixed reference standards of pesticides, including heptachlor, aldrin, endosulfan, dieldrin, endrin, 4,4-DDT, methoxychlor, lindane and cypermethrin, in three replications [19]. Thus, for each fish species, a total of 210 g of muscle tissue was taken for the experiment. Then, the spiked fish samples were retained for 24 h to increase the contact time between the pesticides and the matrix (fish muscle). After 24 h, the household decontamination methods were applied, as stated below, maintaining three replications for each of the decontamination methods. Before spiking with the mixed pesticides solution, it was assured that the muscle tissues were not contaminated with any pesticide residue. The control was untreated. The spiking concentration (500 µg/g) of the mixed reference standards of pesticides was considered based on the available residue levels in water and the biotas of nature [22].

#### 2.3.1. Washing with Running Water (T_1_)

A total of 10 g of the spiked fish samples with three replications were washed for 5 min under running tap water. After wiping with tissue paper, the samples were stored in a freezer until further analysis [23].

#### 2.3.2. Soaking in Stable Normal Water (T_2_)

The pesticides spiked fish samples (10 g) were soaked in an aluminum tray containing 500 mL tap water for 10 min, including three replicates. Then, the treated fish sample trays were preserved in a freezer until further analysis [23].

#### 2.3.3. Soaking in 2% NaCl Solution (T_3_)

A total of 500 mL of 2% NaCl solution was prepared by mixing 10 g of NaCl in 500 mL of water in each aluminum tray, and each of the 10 g samples of the spiked fish muscles were soaked in the tray for 10 min. After that, the treated sample trays were preserved in a freezer until further analysis [24].

#### 2.3.4. Soaking in 2% Vinegar Solution (T_4_)

A total of 500 mL of 2% vinegar solution was prepared by mixing 10 mL of vinegar in 500 mL of water in every aluminum tray, and each of the 10 g samples of the contaminated fishes were soaked in the tray for 10 min. Then, those treated fish sample trays were retained in a freezer until further analysis [23].

#### 2.3.5. Soaking in 0.1% Sodium Bicarbonate Solution (T_5_)

A total of 500 mL of 0.1% sodium bicarbonate solution was formulated by mixing 500 mg of sodium bicarbonate in 500 mL of water in every aluminum tray, and each of the 10 g contaminated spiked fish samples were soaked in the trays for 10 min. Then, the treated fish sample trays were kept in a freezer until further analysis [24].

#### 2.3.6. Soaking in 0.1% Sodium Bicarbonate Solution + 2% NaCl Solution+ 2% Vinegar + Lemon Juice (T_6_)

A mixture of 0.1% sodium bicarbonate and 2% vinegar solution with lemon juice was formulated by mixing 500 mg of sodium bicarbonate, 10 mL of vinegar and the total extracted juice of one lemon in each aluminum tray containing 500 mL of water. Then, the pesticide-spiked fish muscles (10 g) were dipped in those trays for 10 min. After that, the treated sample trays were stored in a freezer until further analysis [24].

All samples were subjected to cleansing with running tap water for 5 min before treatment with T_2_, T_3_, T_4_, T_5_ and T_6_. Each treatment was treated with three replications.

### 2.4. Freezing Storage Duration after the Treatments

After treating the contaminated fish samples with the above-mentioned treatments, the treated trays were preserved in a deep freezer at −40 °C freezing temperature for 14 days before their extraction and clean-up for GC-ECD (gas chromatography with electron capture detector) analysis [25].

### 2.5. Extraction and Clean-Up of the Samples

The QuEChERS (quick, easy, cheap, effective, rugged and safe)-modified method [26] was used for the extraction and clean-up of pesticide residues from the household decontamination method-treated fish matrix. QuEChERS extraction [27] is a ubiquitous extraction and clean-up method for food matrices. The method was performed as described below:

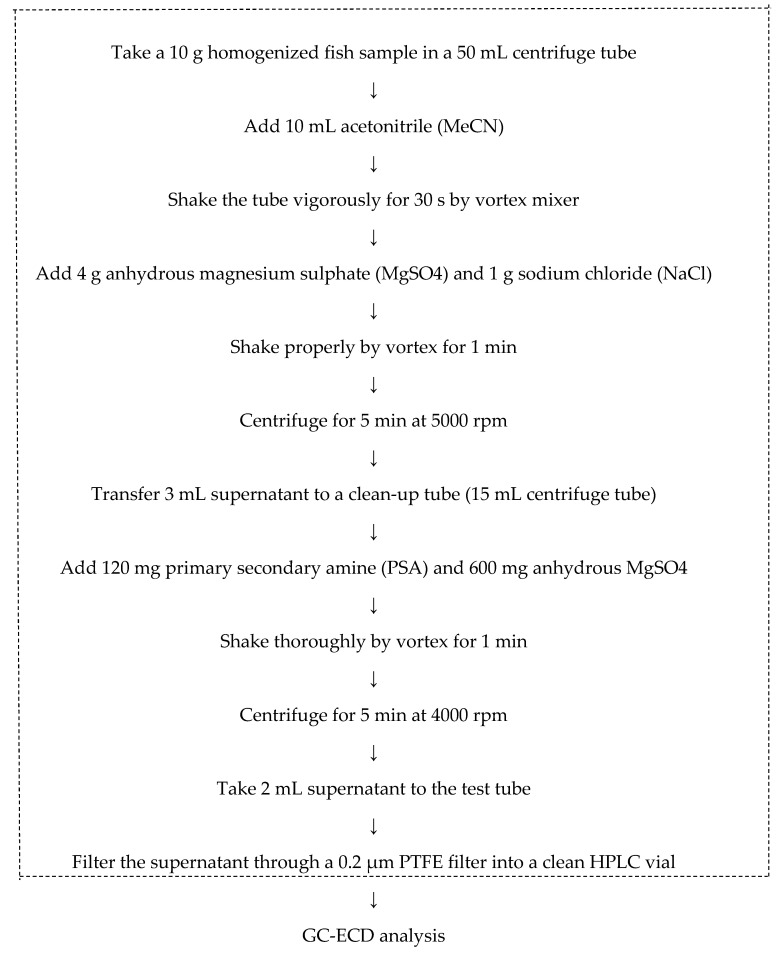



### 2.6. GC-ECD Instrumentation

The GC-ECD analysis was performed in the Pesticide Analysis Lab of the Malaysian Agricultural Research and Development Institute (MARDI), Malaysia. A gas chromatograph (Agilent) equipped with an electron capture detector (ECD) was used to confirm the presence of pesticide residues in the fish samples. A DB-5MS UI capillary column (30 m × 250 μm × 0.25 μm; Agilent, Santa Clara, CA, USA) was used to resolve the target analytes. The maximum temperature of the column was maintained as 325 °C. The GC oven temperature was programmed as follows: initial temperature was set at 80 °C for 1 min, 80 to 200 °C (ramp 50 °C/min) for 0 min, 200 to 240 °C (ramp 5 °C/min) for 0 min, 240 to 310 °C (ramp 12 °C/min) for 8 min and, finally, increased to 325 °C with an equilibration time for 1 min. An autoinjector was used, and the injector temperature was maintained at 280 °C. Ultrapure-grade helium (99.9999%) was used as a carrier gas at a constant flow rate of 1.50 mL/min. The detector (µECD) temperature was 320 °C, with a constant make-up flow of 60 mL/min. The make-up gas used was nitrogen.

### 2.7. Quality Assurance of the Analytical Method

Quality assurance of the analytical method was performed following the established method validation procedures of Prodhan and Alam and Mekonen [26,28] as follows.

#### 2.7.1. Formulation of Mixed Standard Solution from the Reference Pesticides

The reference pesticides mixed with the standard mother solutions were formulated through the addition of lindane, heptachlor, aldrin, endosulfan, dieldrin, DDT, endrin and methoxychlor in an n-hexane solvent at a concentration of 200 mg/L, and then preserved at −20 °C for further application. From the 200 mg/L mother/stock solution of the pesticide-mixed standards, intermediate solutions of 10 mg/l in n-hexane were formulated. After that, the working solutions of 0.50, 1.0, 2.0, 3.0 and 5.0 mg/L of the pesticide-mixed standards were formulated in n-hexane by adding an appropriate amount from the 10 mg/L intermediate mixed standard solution. The working mixed standard solutions were prepared using five individual 5 mL volumetric flasks.

#### 2.7.2. Calibration Curves

For the calibration curve, the matrix-matched standards were formulated by incorporating 100 µL from the mixed reference pesticide standards’ operational solutions of 0.50, 1.0, 2.0, 3.0 and 5.0 mg/L with 900 µL of the blank fish sample extract to reach the fortified concentrations of 0.05, 0.10, 0.20, 0.30 as well as 0.50 mg/L, accordingly. Equally fortified concentrations were also formulated for the n-hexane containing mixed pesticide calibration standards. Whole standard solutions were preserved at −20 °C until their operation.

#### 2.7.3. Assessment of Calibration Curve and Linearity

Five-point calibration curves were prepared by matrix-matched standards and analyzed in triplicate.

#### 2.7.4. Assessment of the Accuracy and Precision Using (%) Recovery of Reference Pesticides

The accuracy of the method was assessed as the percent of the recovery of pesticides from the spiked samples, following the established method of Prodhan and Alam [26], which is represented in Table 1. A total of 10 g of homogenized blank samples of fish were spiked before the extraction operation by the inclusion of a mixed pesticide standard to obtain the final fortification levels of 0.05, 0.25 and 0.50 µg/g. In case of the particular fortification level, five replicates were taken under consideration. After the fortification, the sample was evenly mixed by shaking and then released to settle for a minimum of 30 min, to confirm the adequate contact of the pesticide compounds with the entire matrix. Therefore, the samples were extracted and cleaned up following the modified method reported previously [26]. Precision repeatability/intra-day (RSD_r_) was performed after considering three fortification levels, viz., 0.05, 0.25 and 0.50 µg/g, maintaining 5 replicates within the same day. Precision reproducibility/inter-day (RSD_R_) was demonstrated at two fortification levels of 0.05 and 0.25 µg/g, considering 5 replicates after a 2-month interval [26]. The relative standard deviation (RSD) was calculated using the standard deviation (S) and the mean of the data ($\bar{x}$), following the formula: RSD = (S × 100)/$\bar{x}$.

#### 2.7.5. Assessment of Limit of Detection (LOD) and Limit of Quantification (LOQ)

The limit of detection (LOD) and limit of quantification (LOQ) were assessed following the established method of Prodhan and Alam and Mekonen [26,28], which is demonstrated in Table 2. The method detection limits (MDLs) started from 0.01 mg/Kg for the reference pesticides in fish [29]. The LOQ for all of the selected reference pesticides was settled to 0.05 mg/kg, which was obtained as the acceptable mean (%) recoveries (accuracy) for individual reference pesticides, in the range of 94% to 98%, comparing the acceptable range of (%) recoveries, from 70% to 120%. The precision (RSDr) was less than 10%; acceptable values are RSD < 20%.

### 2.8. Statistical Analysis

The data of the estimated reduction in pesticides (%) were analyzed using SAS 9.4 (SAS Institute Inc., Cary, NC, USA) software by ANOVA analysis, as well as NTSYS software. Data were presented as means ± SD and as a 2D principal component analysis (PCA) graphical cluster. An estimation of the reduction in pesticides (%) was calculated as follows:

Reduction in pesticides (%) = ((Initial deposit − Residues after treatment)/Initial deposit) × 100(1)

## 3. Result

### 3.1. Quality Assurance of the Analytical Method

For the calibration curve at different concentration levels of each reference pesticide, correlation coefficients (R^2^) greater than 0.99 were obtained for the selected nine pesticides. The method was sensitive with LOD and LOQ found as 0.001 and 0.03 µg/g, respectively, except for methoxychlor and cypermethrin (0.01 and 0.03 µg/g). Acceptable recoveries were obtained for the selected pesticides, based on the SANTE guidelines [30,31]. Overall recoveries were 90–98%, with RSDs of less than 14% in the range 0.05–0.5 µg/g using spiked fish samples.

### 3.2. Reduction in Pesticide Residues (%) from African Catfish (Clarias gariepinus) by Various Household Techniques

The reduction (%) in lindane, heptachlor, aldrin, endosulfan, dieldrin, endrin, DDT, methoxychlor and cypermethrin from African catfish (*Clarias gariepinus*) muscles is presented in Table 3 for the treated household techniques. The results revealed that the treatment of fish flesh by dipping in 0.1% sodium bicarbonate solution + 2% vinegar + 2% NaCl solution + lemon juice solution (T_6_) appeared to yield the maximum reduction (%) in those investigated pesticide residues, in contrast to the treatments T_1_, T_2_, T_3_, T_4_ and T_5_. During the treatment of dipping the fish muscle in 0.1% sodium bicarbonate solution + 2% vinegar + 2% NaCl solution + lemon juice solution, the residue level of lindane, heptachlor, aldrin, endosulfan, dieldrin, endrin, DDT, methoxychlor and cypermethrin was reduced to 71–72%, 72–74%, 74–75%, 74–76%, 78–79%, 77–79%, 76–77%, 76.45–77.15% and 78–79%, respectively, which was the significantly (*p* < 0.05) highest reduction compared with the other treatments. The reduction percentages of the residue concentration loads for lindane, heptachlor, aldrin, endosulfan, dieldrin, endrin, DDT, methoxychlor and cypermethrin from African catfish (*Clarias gariepinus*) muscles were observed, in a descending order, as follows: treatment with soaking in 0.1% sodium bicarbonate solution + 2% vinegar + 2% salt solution + lemon juice solution (T_6_) > soaking in 2% vinegar solution (T_4_) > dipping in 0.1% sodium bicarbonate solution (T_5_) > dipping in 2% salt solution (T_3_) > washing with running tap water (T_1_) for 10 min > soaking in stable normal water (T_2_) for 10 min.

### 3.3. Reduction in Pesticide Residues (%) from Snakehead Fish (Channa striata) Muscle by Household Processing Methods

The present study reported that, among the six household treatment processes, dipping the muscle samples in 0.1% sodium bicarbonate solution + 2% vinegar + 2% NaCl solution + lemon juice solution (T_6_) witnessed the best reduction (%) in lindane, heptachlor, aldrin, endosulfan, dieldrin, endrin, DDT, methoxychlor and cypermethrin residues from the snakehead fish (*Channa striata*) muscles. The treatment T_6_ recorded reductions in residue concentration loads of 78–80%, 76–79%, 77–79%, 76–78%, 77–78%, 76–78%, 76–79%, 72–75% as well as 77–78%, respectively, for lindane, heptachlor, aldrin, endosulfan, dieldrin, endrin, DDT, methoxychlor and cypermethrin, which significantly (*p* < 0.05) outperformed the other decontamination processes, viz., T_1_, T_2_, T_3_, T_4_ and T_5_. The reduction percentages of those residue concentrations concerning lindane, heptachlor, aldrin, endosulfan, dieldrin, endrin, DDT, methoxychlor and cypermethrin were obtained as a downward array: the treatment process with soaking in 0.1% sodium bicarbonate solution + 2% vinegar + 2% salt solution + lemon juice solution (T_6_) > dipping in 2% vinegar solution (T_4_) > dipping in 0.1% sodium bicarbonate solution (T_5_) > soaking in 2% salt solution (T_3_) > washing with running tap water (T_1_) for 10 min > soaking in stable normal water (T_2_) for 10 min. This is demonstrated in Table 4.

### 3.4. Reduction in Pesticide Residues (%) from Climbing Perch (Anabus testudineus) Muscle by Household Processing Methods

The present examination evidenced that a decreasing percentage of lindane, heptachlor, aldrin, endosulfan, dieldrin, endrin, DDT, methoxychlor and cypermethrin from the treated muscle samples of climbing perch (*Anabus testudineus*), which is displayed in Table 5, occurred during the six household treatment processes. The results reported that the best reduction in the investigated pesticides was obtained from the muscle samples of *Anabus testudineus* when the treatment process of dipping the fish flesh in 0.1% sodium bicarbonate solution + 2% vinegar + 2% NaCl solution + lemon juice solution (T_6_) was performed, in comparison with the treatments T_1_, T_2_, T_3_, T_4_ and T_5_. The reduction percentage for the concentration of lindane, heptachlor, aldrin, endosulfan, dieldrin, endrin, DDT, methoxychlor and cypermethrin residues during the treatment T_6_ was witnessed as 77–79%, 75–76%, 74–76%, 76–78%, 76–77%, 79–80%, 76–78%, 77–78% as well as 79–80%, respectively, which was more significantly (*p* < 0.05) maximal than the other treatments. The observed reduction percentages’ order of residue concentration loads of lindane, heptachlor, aldrin, endosulfan, dieldrin, endrin, DDT, methoxychlor and cypermethrin were, in descending order, dipping in 0.1% sodium bicarbonate solution + 2% vinegar + 2% salt solution + lemon juice solution (T_6_) > soaking in 2% vinegar solution (T_4_) > dipping in 0.1% sodium bicarbonate solution (T_5_) > soaking in 2% salt solution (T_3_) > washing with running tap water (T_1_) for 10 min > soaking in stable normal water (T_2_) for 10 min.

### 3.5. Reduction in Pesticide Residues (%) from Three-Spot Gourami (Trichogaster trichopterus) Fish Muscle by Household Treatment Processing Methods

The present investigation determined that, among the treated household processes, the treatment process of soaking the muscle samples in 0.1% sodium bicarbonate solution + 2% vinegar + 2% NaCl solution + lemon juice solution (T_6_) also showed an excellent reduction percentage for lindane, heptachlor, aldrin, endosulfan, dieldrin, endrin, DDT, methoxychlor and cypermethrin from three-spot gourami (*Trichogaster trichopterus*) fish muscle. The residual concentration was reduced by the treatment T_6_ as 78–80%, 79–80%, 78–80%, 80–81%, 79–81%, 78–79%, 78–81%, 79–81% and 78–79%, respectively, for lindane, heptachlor, aldrin, endosulfan, dieldrin, endrin, DDT, methoxychlor and cypermethrin, which is more significantly (*p* < 0.05) paramount than other household treatment agents. Thus, the reduction percentage for those examined pesticide residue concentrations were sighted, in descending order, as follows: soaking in 0.1% sodium bicarbonate solution + 2% vinegar + 2% salt solution + lemon juice solution (T_6_) > dipping in 2% vinegar solution (T_4_) > dipping in 0.1% sodium bicarbonate solution (T_5_) > soaking in 2% salt solution (T_3_) > washing with running tap water (T_1_) for 10 min > soaking in stable normal water (T_2_) for 10 min. This is presented in Table 6.

**Figure 1:**
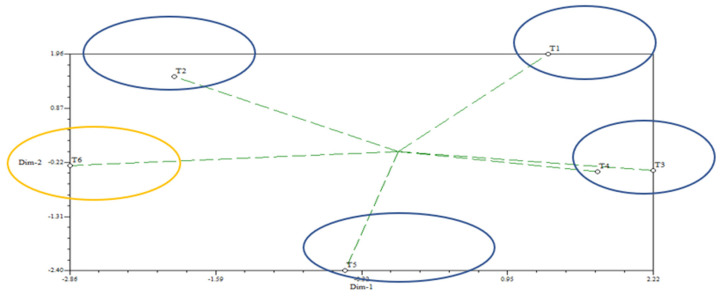
Two−dimensional principal component analysis (PCA) graphical comparison among six treatments’ efficacy, on the basis of remaining pesticide residue concentration in fish muscles after each treatment.

In this present study, the overall reduction (%) for those investigated pesticides, viz., lindane, heptachlor, aldrin, endosulfan, dieldrin, endrin, DDT, methoxychlor and cypermethrin, in the fish muscles of all investigated fish species is demonstrated in Figure 1 using a 2D principal component analysis (PCA) graphical relationship among those household treatments. The distance length of the ‘green-dotted’ eigenvectors from the centroid also revealed the maximal reduction (%) performance of treatment T6 (immersing pesticide-contaminated fish muscle samples in 0.1% sodium bicarbonate solution + 2% vinegar + 2% salt solution + lemon juice solution) for reducing the residue loads of lindane, heptachlor, aldrin, endosulfan, dieldrin, endrin, DDT, methoxychlor and cypermethrin from the fish muscles of those selected fish species.

The current study stated that the treatment of soaking in 0.1% sodium bicarbonate solution + 2% vinegar + 2% salt solution + lemon juice solution (T_6_) showed excellent performance in reducing the pesticide residue concentrations in the muscle samples of all investigated fish species, including African catfish (*Clarias gariepinus*), snakehead fish (*Channa striata*), climbing perch (*Anabus testudineus*) as well as three-spot gourami *(Trichogaster trichopterus)*. Among these investigated fish muscle samples, the reduction percentage order of the residue concentration loads of those pesticides, after being treated with the six household-treatment processes, were as follows: African catfish (*Clarias gariepinus*) < snakehead fish (*Channa striata*) < climbing perch (*Anabus testudineus*) < three-spot gourami *(Trichogaster trichopterus)*.

## 4. Discussion

The described analytical method in this study is an efficient and effective multi-residue analytical method, using gas chromatography coupled with an electron capture detector (GC-ECD) for the determination of the selected nine pesticides in fish. The obtained correlation coefficient (R^2^) values for the calibration curve of each reference pesticide were greater than 0.99 at different concentration levels. Very good accuracy and precision were found for all of the analytes using the analytical method. The adopted method was accurate and met the acceptable criteria, where recoveries were between 80 and 120% and the RSD % values were below 14%. Thus, the practiced method fulfills the requirement set by the SANTE document no. SANTE/11945/2015 for accuracy and precision [32]. The spiked concentration of the present study was lower than the reported concentration (2880 μg/kg) of organochlorine pesticides, such as endosulfan, in the marine biota samples from Klang, Selangor [22].

In the present study, the treatment of soaking fish in the mixture of 0.1% sodium bicarbonate solution + 2% vinegar (acetic acid solution) + 2% salt solution + lemon juice for 10 min, followed by a running tap-water wash for 5 min, was witnessed to reduce lindane, heptachlor, aldrin, endosulfan, dieldrin, endrin, DDT, methoxychlor and cypermethrin residue concentrations by 72–80% in *Channa striata*, 71–79% in *Clarias gariepinus*, 74–80% in *Anabas testudineus* as well as 78–81% in *Trichogaster trichopterus*, which is higher than the reduction percentage rates of fipronil, flubendiamide, chlorantraniliprole, bifenthrin, profenophos, lambda cyhalothrin, imidacloprid as well as betacyfluthrin in field bean pods treated with a mixture of 0.1% sodium bicarbonate solution and 2% vinegar (acetic acid solution) [25].

The treatment of dipping fish in 2% vinegar (acetic acid solution) was found to reduce lindane, heptachlor, aldrin, endosulfan, dieldrin, endrin, DDT, methoxychlor and cypermethrin residue concentrations by 55–60% in *Channa striatus*, 51–56% in *Clarias gariepinus*, 56–61% in *Anabas testudineus* as well as 60–64% in *Trichogaster trichopterus*, which is higher than the reduction percentage values of cypermethrin, profenofos and methomyl pesticides in Chinese kale [13,19], as well as of chlorpyrifos in cauliflower [33]. Contrarily, the removal percentage of lindane, heptachlor, aldrin, endosulfan, dieldrin, endrin, DDT, methoxychlor and cypermethrin is lower than the reduction percentage values of chlorpyrifos (65–79%), chlorothalonil (79.8%), *p*,*p*’-DDT (65.8%) as well as cypermethrin (74%) in Chinese cabbage [14,22]; hexachlorobenzene (HCB), lindane, and DDT in potatoes [7]; and profenofos residues in hot pepper (76.6%), sweet pepper (95.7%) and eggplant (94.6%) [15].

The treatment of soaking fish in 0.1% sodium bicarbonate (NaHCO_3_) solution resulted in reductions in the residual pesticide concentration of lindane, heptachlor, aldrin, endosulfan, dieldrin, endrin, DDT, methoxychlor and cypermethrin of 48–61% in snakehead fish, *Channa striatus*; of 47–52% in African catfish, *Clarias gariepinus*; of 55–60% in climbing perch, *Anabas testudineus*; and of 57–60% in gourami, *Trichogaster trichopterus*. These are higher than the reduction percentage values of dimethoate, chlorpyrifos, quinalphos, profenophos, cyhalothrin and malathion in brinjal [17,34]; of methomyl residues in Chinese kale [13]; of profenophos, flubendiamide, betacyfluthrin, lambda cyhalothrin and bifenthrin in field bean pods [24] as well as of HCB (hexachlorobenzene), lindane and DDT in potatoes [7]. On the other hand, the reduction percentage of lindane, heptachlor, aldrin, endosulfan, dieldrin, endrin, DDT, methoxychlor and cypermethrin by treatment T_5_ (soaking in 0.1% sodium bicarbonate, NaHCO_3_ solution) is lower than the reduction percentage values of chlorpyrifos (85.2%), dimethoate (76.1%), fenitrothion (66.7%) and dichlorvos (98%) in cucumber [35]; cypermethrin (89%) in okra [18]; carbaryl (91%) in Chinese kale [11]; thiabendazole (almost 99%) and phosmet residues in cauliflower [36] as well as 80.00%, 90.40% and 88.20% for dimethoate (80%), primiphos-methyl (90.4%) and malathion (88%) in potatoes [7].

Organic pesticides are mostly soluble in lipids and, thus, can accumulate in the fatty tissues of fish; hence, they are transferred through the food chain. Therefore, the reduction percentage for pesticide residues in fish muscles may differ on the basis of fish species, lipid content and detoxifying enzyme content [37,38], which is evident in our present study.

After 14 days of freezing storage for the treated fish samples, the treatments with household agents were found to reduce the residue loads of lindane, heptachlor, aldrin, endosulfan, dieldrin, endrin, methoxychlor and cypermethrin from raw fish samples. The freezing preservation of contaminated fish at −70 °C was reported to reduce the level of the pesticides by 6–30% [25] because, at freezing temperatures, hydrophobic pesticides interact with the lipid matrices of the samples [39]. Another study of Abou-Arab [40] established that freezing storage around −4 °C can be more effective for decreasing the amount of pesticides residues, as the metabolic activities of cells may still happen, although at a lower rate, in comparison to deep freezing at −70 °C.

During the treatments using household agents, the quality of the fish muscles was not affected by the vinegar or salt due to the osmosis mechanism during the thawing of the treated fish samples with normal water after their freezing storage. Due to the osmosis mechanism, the concentration of vinegar and salt was reduced, which may retain the taste, the flavor as well as the overall quality of the fish muscles. Furthermore, water is the basis for every possible cleaning agent, and it is also a solvent cleanser in itself [20]. Soaking in a diluted salt (sodium chloride) solution is a convenient method to lower the load of the pesticide residues from food commodities. This procedure is recommended as being practical for household use [41]. Organic acids, such as acetic acid, are natural compounds that are generally recognized as safe (GRAS) by the U.S. Food and Drug Administration (U.S. FDA) [42].

Cooking by roasting or deep-frying, contaminated fishes can reduce the concentration levels of endosulfan, heptachlor, malathion, chlorpyrifos, bifenthrin, deltamethrin and fenoxycarb by up to 93%, but not 100% [25]. So, the present study recommends that, before cooking, contaminated fishes should be treated by soaking them in a mixture of 0.1% sodium bicarbonate solution, 2% vinegar (acetic acid solution), 2% salt solution and lemon juice for 10 min, followed by a running tap-water wash for 5 min. Further preservation in a freezer and cooking may reduce the levels of pesticides even more.

## 5. Conclusions

This study has determined the most efficient household treatment for the lessening of pesticide residues from raw fish muscles before cooking. To our knowledge, this is the first study looking into the reduction in pesticide residue in precooked fish by household treatments. It is clear from this study that the level of pesticide residues in fish muscle samples is influenced noticeably by the method of soaking in 0.1% sodium bicarbonate solution + 2% vinegar + 2% salt solution with lemon juice after freezing for 14 days at −40 °C. This study also indicates the importance of household decontamination techniques to reduce pesticide residues from raw fish items to ensure consumers’ utmost food safety, as they are easily affordable for all classes of consumers. However, more studies are required on other household decontamination techniques to reduce pesticide residues from other raw fish species, as the degree of reduction differs with the nature of the pesticide molecules, the variety of the food commodity, the treatment types and the preparation methods for the product.

## Figures and Tables

**Table 1:** Mean recovery (%) (accuracy) and RSD (%) (precision) of the selected reference pesticides in the fish matrix at different fortification levels.

Reference Pesticides Standards	% Recovery of Reference Pesticides (*n =* 5) (Intra-Day)						% Recovery of Reference Pesticides (*n =* 5) (Inter-Day)			
	0.05 (µg/g)		0.25 (µg/g)		0.50 (µg/g)		0.05 (µg/g)		0.25 (µg/g)	
	Mean (%)	RSD_r_ (%)	Mean (%)	RSD_r_ (%)	Mean (%)	RSD_r_ (%)	Mean (%)	RSD_R_ (%)	Mean (%)	RSD_R_ (%)
Lindane	95.85	9.66	94.33	6.01	90.96	11.45	90.05	2.40	89.46	2.63
Heptachlor	94.80	5.61	90.99	6.90	94.30	4.70	93.47	2.98	92.85	2.52
Aldrin	96.52	7.90	96.68	13.83	96.10	11.58	94.52	3.78	93.08	4.96
Endosulfan	97.44	5.13	97.41	11.62	96.20	10.38	94.63	4.77	96.61	5.10
Dieldrin	98.78	5.16	93.97	5.17	95.23	10.98	95.78	2.70	93.17	3.74
DDT	97.75	4.50	91.30	5.13	97.18	3.36	95.46	2.03	95.30	2.20
Endrin	97.87	4.60	93.04	8.43	96.83	11.95	96.27	2.79	92.03	2.97
Methoxychlor	97.24	5.46	93.73	8.21	98.60	10.01	96.63	4.11	93.32	4.75
Cypermethrin	98.19	8.80	97.05	8.29	93.65	10.44	94.19	3.12	94.84	4.22

**Table 2:** Retention time (RT), limit of detection (LOD), limit of quantification (LOQ) and coefficient of determination (R^2^) of the selected reference pesticides in the fish matrix at different fortification levels.

Reference Pesticides	RT	LOD (µg/g)	LOQ (µg/g)	R^2^ (Calibration Curve)
Lindane	5.97	0.012	0.05	0.9977
Heptachlor	7.05	0.007	0.05	0.9962
Aldrin	7.69	0.006	0.05	0.9971
Endosulfan	9.24	0.009	0.05	0.9969
Dieldrin	9.87	0.004	0.05	0.9973
DDT	10.65	0.005	0.05	0.9959
Endrin	10.39	0.006	0.05	0.9938
Methoxychlor	13.13	0.010	0.05	0.9940
Cypermethrin	15.90	0.010	0.05	0.9982

**Table 3:** Reduction in pesticide residues (%) in *Clarias gariepinus* by household treatments.

Pesticides	Measured Residue (µg/g) in Untreated Control (*n =* 3)	Residue Reduction (%) after Each Treatment (*n =* 3)					
		T_1_	T_2_	T_3_	T_4_	T_5_	T_6_
Lindane	0.479 ± 0.001	15–18	11–13	47–49	51–53	47–49	71–72
Heptachlor	0.484 ± 0.001	16–18	12–14	49–50	52–53	49–50	72–74
Aldrin	0.483 ± 0.016	15–17	10–14	49–50	54–55	49–50	74–75
Endosulfan	0.488 ± 0.014	16–20	11–15	50–51	53–55	49–50	74–76
Dieldrin	0.495 ± 0.004	16–18	13–16	49–51	55–56	50–51	78–79
Endrin	0.491 ± 0.006	14–16	11–17	51–52	55–56	51–52	77–79
DDT	0.489 ± 0.010	15–18	12–15	49–50	53–55	49–50	76–77
Methoxychlor	0.488 ± 0.0017	16–17	15–16	48–49	53–54	48–50	76–77
Cypermethrin	0.492 ± 0.002	16–19	16–20	50–51	54–56	50–51	78–79

Here, T_1_ = treatment of washing with running tap water; T_2_ = treatment of soaking in stable normal water; T_3_ = treatment of dipping in 2% salt solution; T_4_ = treatment of soaking in 2% vinegar solution; T_5_ = treatment of soaking in 0.1% sodium bicarbonate solution; T_6_ = treatment of dipping in 0.1% sodium bicarbonate solution + 2% vinegar + 2% salt solution + lemon juice solution. The residue reduction (%) is displayed as a range, using the mean ± SD.

**Table 4:** Reduction in pesticide residues (%) in *Channa striata* by the six household treatment techniques.

Pesticides	Measured Residue (µg/g) in Untreated Control (*n =* 3)	Residue Reduction (%) after Each Treatment (*n =* 3)					
		T_1_	T_2_	T_3_	T_4_	T_5_	T_6_
Lindane	0.487 ± 0.002	24–30	13–15	37–38	55–56	58–60	78–80
Heptachlor	0.484 ± 0.004	26–33	16–18	37–41	55–58	48–51	76–79
Aldrin	0.491 ± 0.001	28–31	22–23	41–43	58–60	55–59	77–79
Endosulfan	0.493 ± 0.003	33–35	18–20	44–45	58–59	59–61	76–78
Dieldrin	0.492 ± 0.003	35–37	15–18	45–46	58–60	50–53	77–78
Endrin	0.494 ± 0.002	28–33	12–16	51–55	59–60	55–57	76–78
DDT	0.491 ± 0.001	29–30	15–16	51–53	60–62	54–57	76–79
Methoxychlor	0.488 ± 0.003	33–34	13–14	50–51	58–60	55–57	72–75
Cypermethrin	0.493 ± 0.003	32–33	14–15	53–55	58–60	55–57	77–78

Here, T_1_ = treatment of washing with running tap water; T_2_ = treatment of soaking in stable normal water; T_3_ = treatment of dipping in 2% salt solution; T_4_ = treatment of soaking in 2% vinegar solution; T_5_ = treatment of soaking in 0.1% sodium bicarbonate solution; T_6_ = treatment of dipping in 0.1% sodium bicarbonate solution + 2% vinegar + 2% salt solution + lemon juice solution. The residue reduction (%) is displayed as a range, using the mean ± SD.

**Table 5:** Reduction in pesticide residues (%) in *Anabus testudineus* by the six household treatments.

Pesticides	Measured Residue (µg/g) in Untreated Control (*n =* 3)	Residue Reduction (%) after each Treatment (*n =* 3)					
		T_1_	T_2_	T_3_	T_4_	T_5_	T_6_
Lindane	0.482 ± 0.003	35–37	15–19	44–45	57–58	57–58	77–79
Heptachlor	0.479 ± 0.002	35–36	17–20	42–43	56–57	55–57	75–76
Aldrin	0.486 ± 0.004	34–36	16–20	44–45	58–59	55–56	74–76
Endosulfan	0.487 ± 0.003	35–36	18–19	45–47	59–60	56–59	76–78
Dieldrin	0.488 ± 0.002	37–39	19–20	46–47	59–60	56–57	76–77
Endrin	0.489 ± 0.003	38–41	18–19	46–48	60–61	58–59	79–80
DDT	0.486 ± 0.005	38–39	19–21	45–46	57–59	58–59	76–78
Methoxychlor	0.481 ± 0.005	37–38	20–22	45–47	58–59	56–58	77–78
Cypermethrin	0.490 ± 0.002	38–39	19–22	46–47	58–60	58–60	79–80

Here, T_1_ = treatment of washing with running tap water; T_2_ = treatment of soaking in stable normal water; T_3_ = treatment of dipping in 2% salt solution; T_4_ = treatment of immersing in 2% vinegar solution; T_5_ = treatment of soaking in 0.1% sodium bicarbonate solution; T_6_ = treatment of immersing in 0.1% sodium bicarbonate solution + 2% vinegar + 2% salt solution + lemon juice solution. The residue reduction (%) is displayed as a range, using the mean ± SD.

**Table 6:** Reduction in pesticide residues (%) from three-spot gourami *(Trichogaster trichopterus)* by the six household treatments.

Pesticides	Measured Residue (µg/g) in Untreated Control (*n =* 3)	Residue Reduction (%) after Each Treatment (*n =* 3)					
		T_1_	T_2_	T_3_	T_4_	T_5_	T_6_
Lindane	0.490 ± 0.002	34–36	15–17	53–55	60–61	57–60	78–80
Heptachlor	0.490 ± 0.003	35–36	17–18	54–55	62–63	58–59	79–80
Aldrin	0.492 ± 0.003	35–37	16–19	56–57	61–63	59–60	78–80
Endosulfan	0.492 ± 0.002	36–38	17–19	55–57	62–64	58–60	80–81
Dieldrin	0.493 ± 0.004	36–38	15–16	57–58	63–64	57–59	79–81
Endrin	0.492 ± 0.004	37–38	16–18	56–58	62–63	58–60	78–79
DDT	0.493 ± 0.003	36–38	16–17	56–58	61–62	58–59	78–81
Methoxychlor	0.494 ± 0.002	37–38	17–18	55–56	60–62	58–60	79–81
Cypermethrin	0.492 ± 0.004	36–39	18–20	56–58	60–61	59–60	78–79

Here, T_1_ = treatment of washing with running tap water; T_2_ = treatment of soaking in stable normal water; T_3_ = treatment of dipping in 2% salt solution; T_4_ = treatment of immersing in 2% vinegar solution; T_5_ = treatment of soaking in 0.1% sodium bicarbonate solution; T_6_ = treatment of immersing in 0.1% sodium bicarbonate solution + 2% vinegar + 2% salt solution + lemon juice solution. The residue reduction (%) is displayed as a range, using mean ±SD.

## Data Availability

Data are available for financial support of this study through the National Agricultural Technology Program-2 (NATP-II), Bangladesh, project memo number NATP-2/PIU-BARC-44/2017/1662 (54).
